# Patterns and processes underlying understory songbird communities in southern China

**DOI:** 10.1002/ece3.11446

**Published:** 2024-06-06

**Authors:** Fangyuan Liu, Xiaoping Yu, Xianli Che, Qiang Zhang, Alexandra Ashley Grossi, Min Zhang, Zhengzhen Wang, Fasheng Zou

**Affiliations:** ^1^ College of Life Sciences Shaanxi Normal University Xi'an China; ^2^ Guangdong Key Laboratory of Animal Conservation and Resource Utilization, Guangdong Public Library of Wild Animal Conservation and Utilization, Institute of Zoology Guangdong Academy of Sciences Guangzhou China; ^3^ Key Laboratory for Biodiversity Science and Ecological Engineering, College of Life Sciences Beijing Normal University Beijing China

**Keywords:** community structure, distribution pattern, diversity, functional traits, southern China, understory birds

## Abstract

Understory bird communities, especially those comprising insectivores, are highly sensitive to forest loss and fragmentation. Currently, there is little knowledge regarding the large‐scale diversity patterns of understory bird communities, particularly in Eastern Asia. Consequently, we aimed to identify the distribution patterns of understory birds in southern China and the factors underlying these patterns. We analysed the diversity distribution patterns of taxonomic and functional α and β diversity for understory Passeriformes birds in southern China utilising cluster and ordination analyses. Subsequently, we analysed the effects of geographic distance, annual mean temperature, annual temperature range, annual mean precipitation, and annual precipitation range on diversity distribution patterns. In total, 9282 individuals belonging to 11 orders, 48 families, and 297 species were captured over 98,544 net hours, with Alcippeidae being the most abundant family in southern China. The understory bird communities of the 25 sites were categorised into six sub‐regions of the Oriental Realm (Indo‐Malayan Realm). The pattern in the distribution of taxonomic and functional β‐diversity of understory birds in southern China was consistent with zoogeographical regionalisation. Three distinct geographical groups were identified: Group 1 was located in the Min‐Guang Coast and Hainan sub‐regions; Group 2 was located in the East Hilly Plain, Southwest Mountains, and Western Mountains and Plateaus sub‐regions; and Group 3 was located in the Southern Yunnan Mountain subregion. The most critical factors related to the distribution patterns of β‐diversity were geographical distance, annual mean temperature, and annual temperature range. Our results showed that the understory bird communities of the Southwest Mountain, East Hilly Plain, and Western Mountains, and Plateaus sub‐regions were similar, as were those of the Min‐Guang Coast and Hainan sub‐regions. Our results underscore the joint roles of distance, temperature, and historical evolution in understory bird communities.

## Graphical abstract

In this study, we investigated the distribution patterns of understory bird communities in southern China, elucidating the factors influencing their diversity. Our research fills a knowledge gap in understanding large‐scale diversity patterns in songbird communities, thereby making a substantial contribution to the literature on avian ecology and evolution.

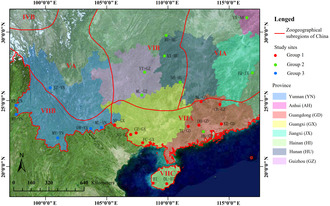

## 1 INTRODUCTION

Forest loss and environmental degradation pose a significant threat to understory bird communities worldwide (Betts et al., 2017; Stouffer et al., 2020). Additionally, understory avian species have limited mobility and weak or seasonal territoriality, which make dispersal difficult for most species; therefore, understory birds heavily rely on forest ecosystems (Moore et al., 2008; Sieving et al., 1996; Visco et al., 2015). This dependence is exacerbated by species that demonstrate an aversion to cross open areas (Stratford & Robinson, 2005). The fragmentation of forest habitats adversely impacts forest‐dependent understory species, as well as forest interior species (Askins et al., 1990; Newmark, 1991; Stouffer et al., 2009). This impact is particularly notable on understory insectivorous birds (Powell, Cordeiro, & Stratford, 2015; Powell, Wolfe, et al., 2015; Stratford & Stouffer, 1999, 2015). The scarce mobility and limited seasonal territoriality of understory birds restrict their movement between forest fragments (Develey & Stouffer, 2001; Laurance, 2004; Moore et al., 2008), making them vulnerable to local extinction in fragmented habitats (Lens et al., 2002; Powell, 2013). Overall, fragmentation influences survival ratio, recruitment, and population growth (Zhang, 2011). Studies on the factors that determine understory birds have mostly been conducted in South American populations, and only a few have been conducted in Asian populations (Sreekar et al., 2015). Inadequate sampling may ignore the influence of large regional differences. Hence, some study results in a certain area of understory birds are inapplicable to another area. It is significant for us to expand the study scale as large as possible.

Previous studies of understory bird communities in China have primarily focused on community composition. The understory bird communities in Guangdong and Hainan mostly belong to the Timaliidae family (Passeriformes) (Zhang, 2011; Zou & Chen, 2004). Insectivores accounted for 70% and 77.6% of total species and total captures in Hainan, respectively. The most prevalent species was the Huet's  Fulvetta (*Alcippe hueti*), accounting for 37.9% and 29.5% of the total captures in the primary and secondary tropical mountain rainforest, respectively (Zou & Chen, 2004). In contrast to Hainan, in the Yunnan province, higher species richness and lower abundance were observed, with the most prevalent species being the Sliver‐breasted Broadbill (*Serilophus lunatus*), accounting for 12.7% of the total species (Huang & Zou, 2010).

To ensure the efficient conservation of understory birds, it is essential to possess knowledge not only regarding community composition and distribution patterns but also a comprehensive understanding of the various environmental factors that influence them. Many studies have shown that the horizontal, vertical, or temporal distribution patterns of diversity between local species communities were common in understory birds (Montaño‐Centellas et al., 2020; Sayer et al., 2017; Sun et al., 2022; Zhang et al., 2020). Additionally, the α diversity of understory birds has previously been associated with temperature, microclimate, and precipitation (Meng et al., 2021; Neate‐Clegg et al., 2021; Powell, Cordeiro, & Stratford, 2015; Powell, Wolfe, et al., 2015; Visco et al., 2015). Spatial variation in bird assemblage structure was also significantly correlated with environmental and topographic variables but not strongly related to spatial variables (Menger et al., 2017).

In this study, we focused on understory bird communities in southern China. Our primary research objectives were to investigate the geographical variation in bird communities between regions and identify the traits and environmental factors underlying the community differences. Furthermore, we aimed to devise comprehensive strategies for the conservation of the entire range of understory bird communities, taking into account any existing spatial‐scale patterns.

## 2 METHODS

### 2.1 Study area

**FIGURE 1:**
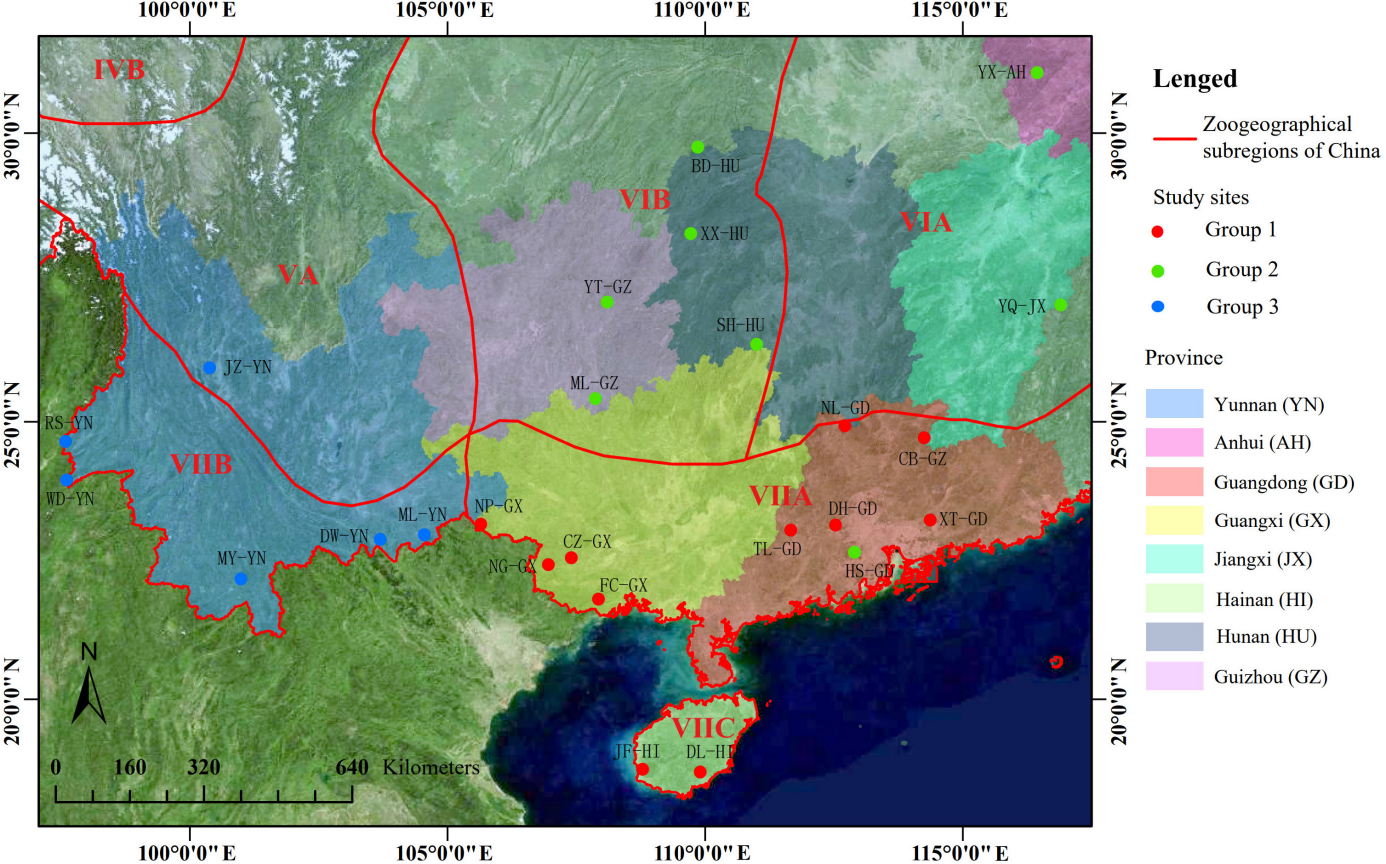
Geographical distribution of 25 understory Passeriformes bird communities. The Roman numerals represent different zoogeographical subregions (see Table 1), dots represent sampling sites.

Our study consisted of 25 sampling sites located in southern China, belonging to six zoogeographical sub‐regions of the Oriental Realm (Table 1 and Figure 1). Among of them, the Heshan Guangdong (HS‐GD) was the only sampling site located in an artificial forest. We realised that one artificial forest sampling was a weakness. We decided to include it in order to encourage more research on the understory of artificial forests in the future and found differences in understory birds between natural forests and artificial forests. Most sites in this study, particularly the western and southern sites, were global diversity hotspots.

**TABLE 1:** Sampling sites and their corresponding zoogeographical sub‐regions.

Sampling site name	Latitude	Longitude	Province	Sampling year	Zoogeographical sub‐region^a^
Jizushan (JZ‐YN)	25.95756	100.39220	Yunnan	2011;2012	Southwest Mountain subregion (VA)
Yuexi (YX‐AH)	31.01660	116.44572	Anhui	2015	East Hilly Plain subregion (VIA)
Yanquan (YQ‐JX)	27.06176	116.90524	Jiangxi	2011	
Nanling (NL‐GD)	24.93197	112.71608	Guangdong	2016;2018;2019	
Badagongshan (BD‐HU)	29.76598	109.86615	Hunan	2012;2013	Western Mountains and Plateaus subregion (VIB)
Xiaoxi (XX‐HU)	28.28408	109.73034	Hunan	2022	
Shunhuangshan (SH‐HU)	26.37122	111.01002	Hunan	2018;2019	
Yuntaishan (YT‐GZ)	27.10663	108.11256	Guizhou	2011;2012	
Maolan (ML‐GZ)	25.41399	107.87374	Guizhou	2010	
Napo (NP‐GX)	23.18417	105.65333	Guangxi	2014	Min‐Guang Coast subregion (VIIA)
Nonggang (NG‐GX)	22.45702	106.97023	Guangxi	2005;2006	
Chongzuo (CZ‐GX)	22.57944	107.41361	Guangxi	2016	
Fangchenggang (FC‐GX)	21.82722	107.93944	Guangxi	2014	
Tongledashan (TL‐GD)	23.07466	111.66636	Guangdong	2014;2015	
Dinghushan (DH‐GD)	23.15937	112.53817	Guangdong	2015;2017–2019	
Heshan (HS‐GD)	22.67679	112.89830	Guangdong	2003;2004	
Chebaling (CB‐GD)	24.72464	114.25709	Guangdong	2006–2009;2012–2016	
Xiangtoushan (XT‐GD)	23.25828	114.37135	Guangdong	2017–2019	
Rongshuwang (RS‐YN)	24.66398	97.592117	Yunnan	2013	Southern Yunnan Mountain subregion (VIIB)
Wudian (WD‐YN)	23.96990	97.615583	Yunnan	2013	
Mengyang (MY‐YN)	22.20222	100.99588	Yunnan	2012;2013	
Daweishan (DW‐YN)	22.90437	103.69877	Yunnan	2011;2012	
Malipo (ML‐YN)	22.99194	104.55528	Yunnan	2016	
Jianfengling (JF‐HI)	18.71010	108.79072	Hainan	2000–2002	Hainan subregion (VIIC)
Diaoluoshan (DL‐HI)	18.66238	109.90641	Hainan	2007;2008	

^a^ Zoogeographical sub‐region based on Zhang (1999).

### 2.2 Data collection

The understory bird data used for our analysis were assembled from the literature as well as the surveys conducted by the authors. Understory bird data for nine sampling sites were obtained from published literature: Nonggang Guangxi (NG‐GX) (Jiang, 2007), Yuntai Guizhou (YT‐GZ) (Luo et al., 2013), Mengyang Yunnan (MY‐YN) (Huang & Zou, 2010), Daweishan Yunnan (DW‐YN) (Su et al., 2014), Jizushan Yunnan (JZ‐YN) (Su et al., 2014), Heshan Guangdong (HS‐GD) (Zou & Yang, 2005), Nanling Guangdong (NL‐GD) (Zhang et al., 2018), Chebaling Guangdong (CB‐GD) (Zhang et al., 2018), and Jianfengling Hainan (JF‐HI) (Zou & Chen, 2004). To the best of our knowledge, this list encompasses all the studies conducted on understory birds in China. Data from the remaining 16 sampling sites were obtained from surveys conducted between 2000 and 2022. Birds at each sampling site constituted a distinct understory community. The capture of all understory birds was carried out using mist nets with a 36 mm mesh and 12 m length. Mist nets were deployed from 6:00 a.m. to 18:00 p.m. on days characterised by sunny weather conditions and minimal wind disturbance. The nets were checked every 2 h, and captured birds were recorded and immediately released. The taxonomic classification and nomenclature used for species identification adhered to the guidelines set forth by the International Ornithological Committee World Bird List (Gill et al., 2022). Except for Passeriformes, most orders were represented by only one or a few captured individuals belonging to one or a few families. For instance, Caprimulgiformes and Charadriiformes each included a single bird, whereas five individuals belonging to the Coraciiformes family represented one species. Therefore, we excluded other species and specifically analysed the Passeriformes family.

Functional trait data included morphological features reflecting flying ability (hand‐wing index and body mass) and ecological features reflecting adaptability (migratory behaviour, territoriality, breeding system, diet, and foraging behaviour). These trait data were obtained from published reports (Tobias et al., 2022; Tobias & Pigot, 2019). Geographical distance refers to the straight‐line distance between two sites.

According to a previous study (Young et al., 1998), we selected the four most relevant and most commonly used environmental variables for bird distribution and movement studies. These include annual mean temperature (AMT), annual temperature range (ATR), annual mean precipitation (AMP), and annual precipitation range (APR). We retrieved the original data on environmental variables from the National Earth System Science Data Center database (Tables S1 and S2; modified from Peng et al., 2019) and extracted the monthly temperature and monthly precipitation data for the sampling year at the sampling point using ArcGIS 10.0 software (ESRI, 2010). Subsequently, we calculated AMT, AMP, ATR, and APR, which represented the difference between the highest and lowest monthly values within the sampling year.

### 2.3 Statistical analysis

To comprehensively assess the characteristics of understory Passeriforme communities in southern China, we identified the community composition at each site in terms of taxonomic composition (main family), migration behaviour, territoriality, breeding system, diet type, and foraging behaviour.

Three species diversity indexes were calculated: Shannon–Winner index, Simpson's index, and Pielou evenness index as taxonomic α‐diversity in the “diversity” function of the “Vegan package”. Two functional diversity indexes including functional evenness index (FEve) and functional divergence index (FDiv) were calculated based on four traits, namely hand‐wing index, body mass, diet, and foraging behaviour. These two indicators were also calculated using the “dbFD” function in the “FD” package (Laliberté et al., 2014). We used capture individuals to evaluate whether there was a significant difference in the taxonomic α‐diversity indexes between groups.

Taxonomic β‐diversity was measured using the Bray–Curtis dissimilarity index and calculated employing the “beta. pair. Bound” function of the “betapart” package (Baselga et al., 2022). Functional β‐diversity was measured using the β mean nearest taxon distance (β MNTD) with the “comdistnt” function of the “Picante” package (Oksanen et al., 2022). Permutational multivariate analysis of variance (PERMANOVA) was used to test the statistical significance of differences between distinct groups in the taxonomic and functional β‐diversity analysis, which was performed using the “adonis 2” function in the “Vegan” package.

To determine the distribution pattern of understory bird communities, we deployed the unweighted pair group method algorithm (UPGMA) hierarchical clustering analysis to categorise the 25 understory Passeriformes bird communities into different subgroups. We also utilised a principal coordinate analysis (PCoA) to visualise and validate the coherence of the resulting clusters. The clustering results were projected onto geographical locations. UPGMA and PCoA analysis were both performed using the “hclust” function in “ade4” and the “cmdscale” function in the “Vegan” package, respectively (Bougeard & Dray, 2018; Oksanen et al., 2022). Statistical analysis of variance was performed using SPSS 26 (IBM Corp., 2012).

Furthermore, we used a Mantel test to analyse the correlations between environment variable matrices and taxonomic and functional β‐diversity using the “mantel” function of the “Vegan” package. Multiple regression was applied to dissimilarity matrices (MRM) to ascertain the relative importance of each environmental variable using the “MRM” function of the “ecodist” package (Goslee & Urban, 2007). All analyses, unless otherwise mentioned, were performed in the R 4.2.0 environment (R Core Team, 2022).

## 3 RESULTS

### 3.1 Species composition

A total of 8893 individuals belonging to the order Passeriformes, representing 36 families and 251 species, were included in the analysis. Twenty‐two species were listed as Chinese State Key Protected Wild Animals, accounting for 8.7% of the total species captured. Two species were on the IUCN Red List (IUCN, 2021): Brown‐chested Jungle Flycatcher (*Cyornis brunneatus*; vulnerable) and Chinese Grassbird (*Graminicola striatus*; near threatened).

**FIGURE 2:**
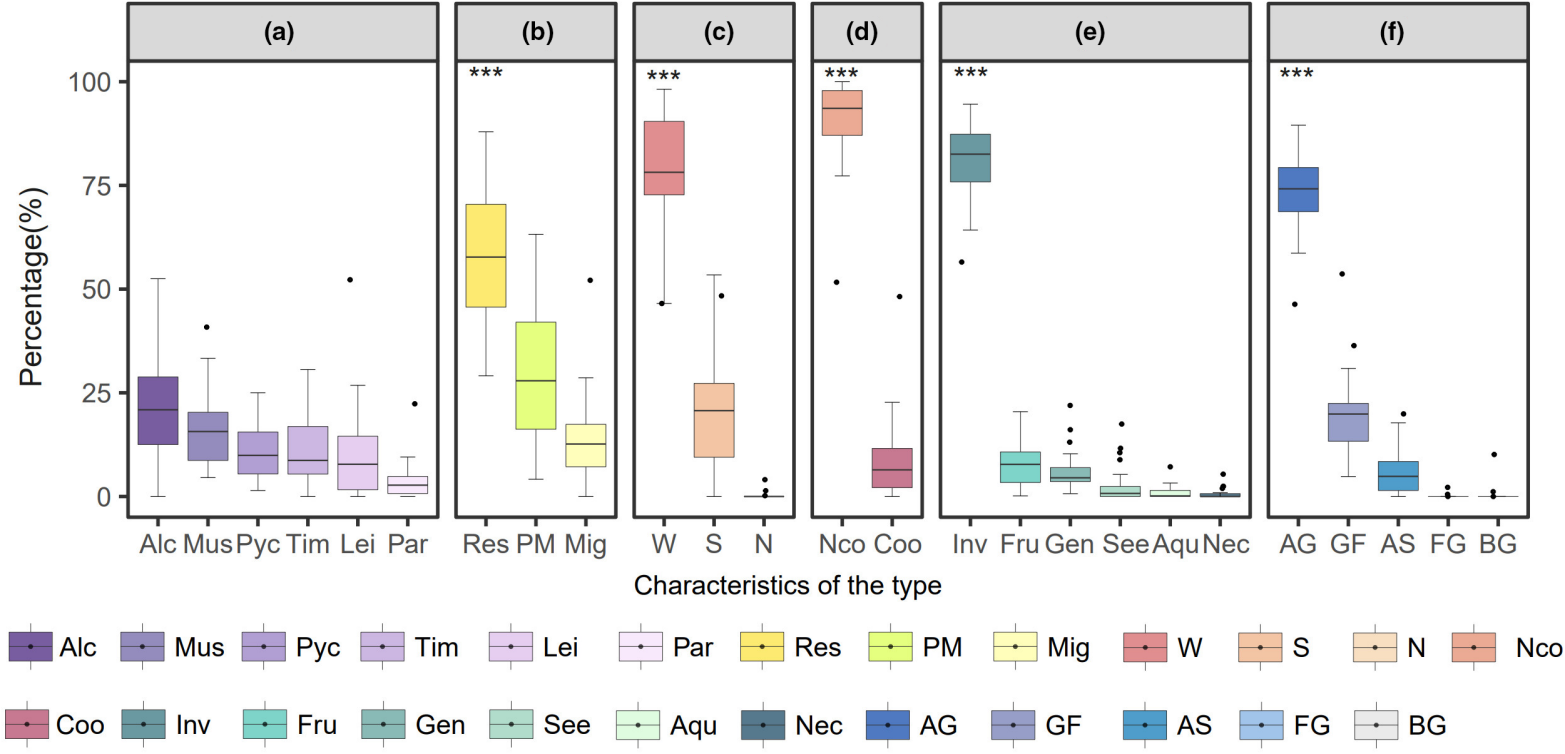
Community compositions of understory Passeriformes bird communities: family (a), migration behaviour (b), territoriality (c), breeding system (d), diet type (e), and foraging behaviour (f). Letter code: Alc (Alcippeidae), Mus (Muscicapidae), Pyc (Pycnonotidae), Tim (Timaliidae), Lei (Leiothrichidae), Par (Paridae); Res (resident), PM (partially migratory), Mig (migratory); W (weak territoriality), S (strong territoriality), N (none territoriality); Nco (Noncooperative breeding), Coo (cooperative breeding); Inv (invertebrates), Fru (frugivores), Gen (generalist), See (seeds), Aqu (aquatic animals), Nec (nectarivores); AG (arboreal gleaning), GF (ground foraging), AS (aerial sallying), FG (foraging generalist), BG (bark gleaning). We only show the first six families in this figure. The boxeplots were sorted by median from largest to smallest in each type. “***”: indicate statistically significant differences at *p* < .001 level.

The understory Passeriformes bird communities in southern China based on mean species richness mainly consisted of Alcippeidae (22.1 ± 2.6%), with a few communities being dominated by Muscicapidae (15.9 ± 1.7%), Timaliidae (11.6 ± 1.6%), Leiothrichidae (10.5 ± 2.3%), and Pycnonotidae (10.0 ± 1.2%; Figure 2a). Autochthonous species were the dominant group in natural forests (57.9 ± 3.3%), while migratory birds accounted for 13.6 ± 2.2% (Figure 2b). The proportion of weakly territorial birds was significantly higher (78.3 ± 3.1%) than that of strongly territorial birds (21.2 ± 3.1%) and non‐territorial birds (0.3 ± 0.1%; Figure 2c). Of note, the breeding system type was primarily non‐cooperative (90.4 ± 2.1%; Figure 2d). Most bird species fed on invertebrates (81.3 ± 2.0%; Figure 2e), with arboreal gleaning species (73.0 ± 2.0%) being the main foraging behaviour (Figure 2f).

### 3.2 Bird diversity and zoogeographical regions

**FIGURE 3:**
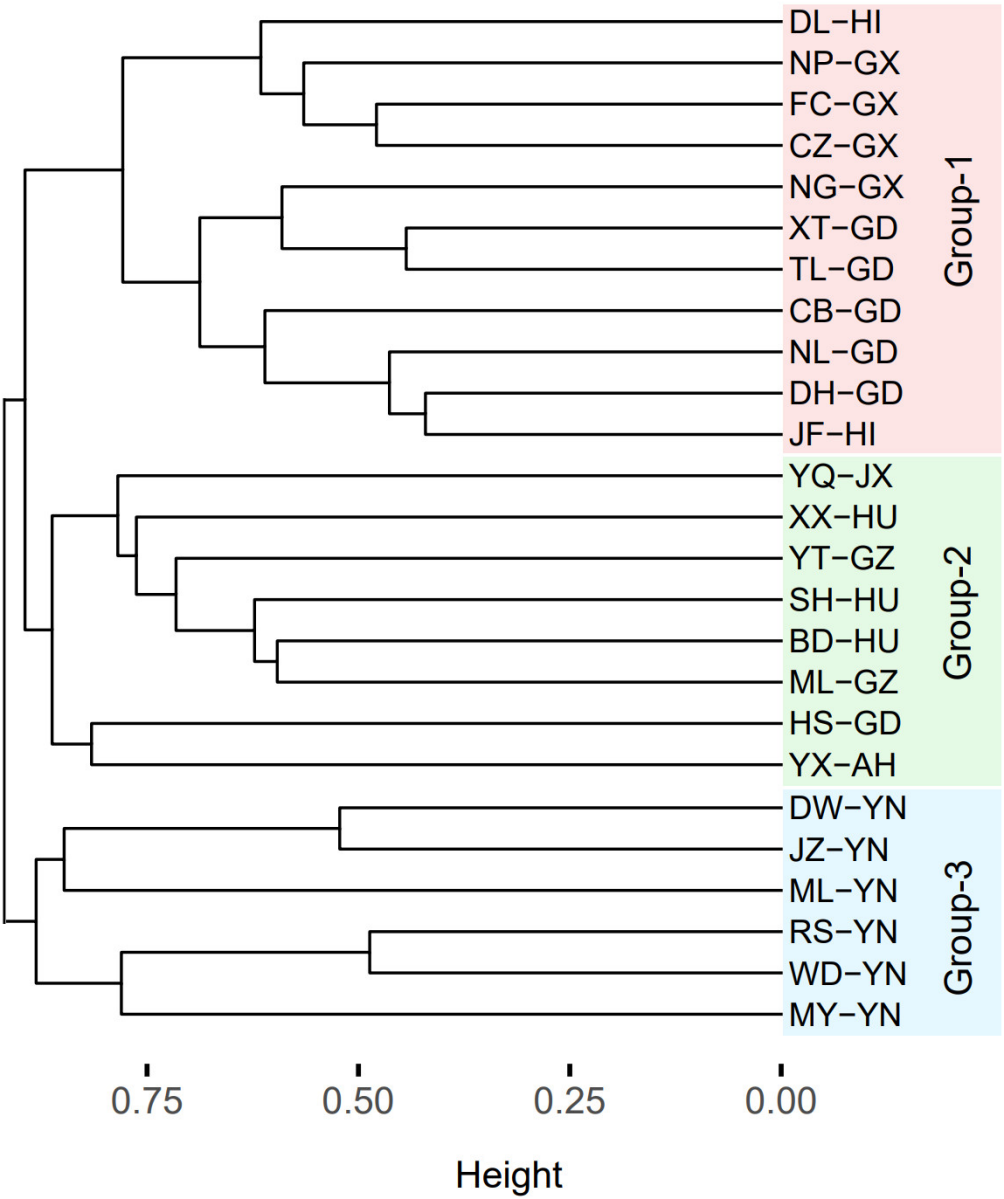
Hierarchical cluster analysis of 25 understory Passeriform communities in southern China. The cluster tree was established according to taxonomic β‐diversity.

**FIGURE 4:**
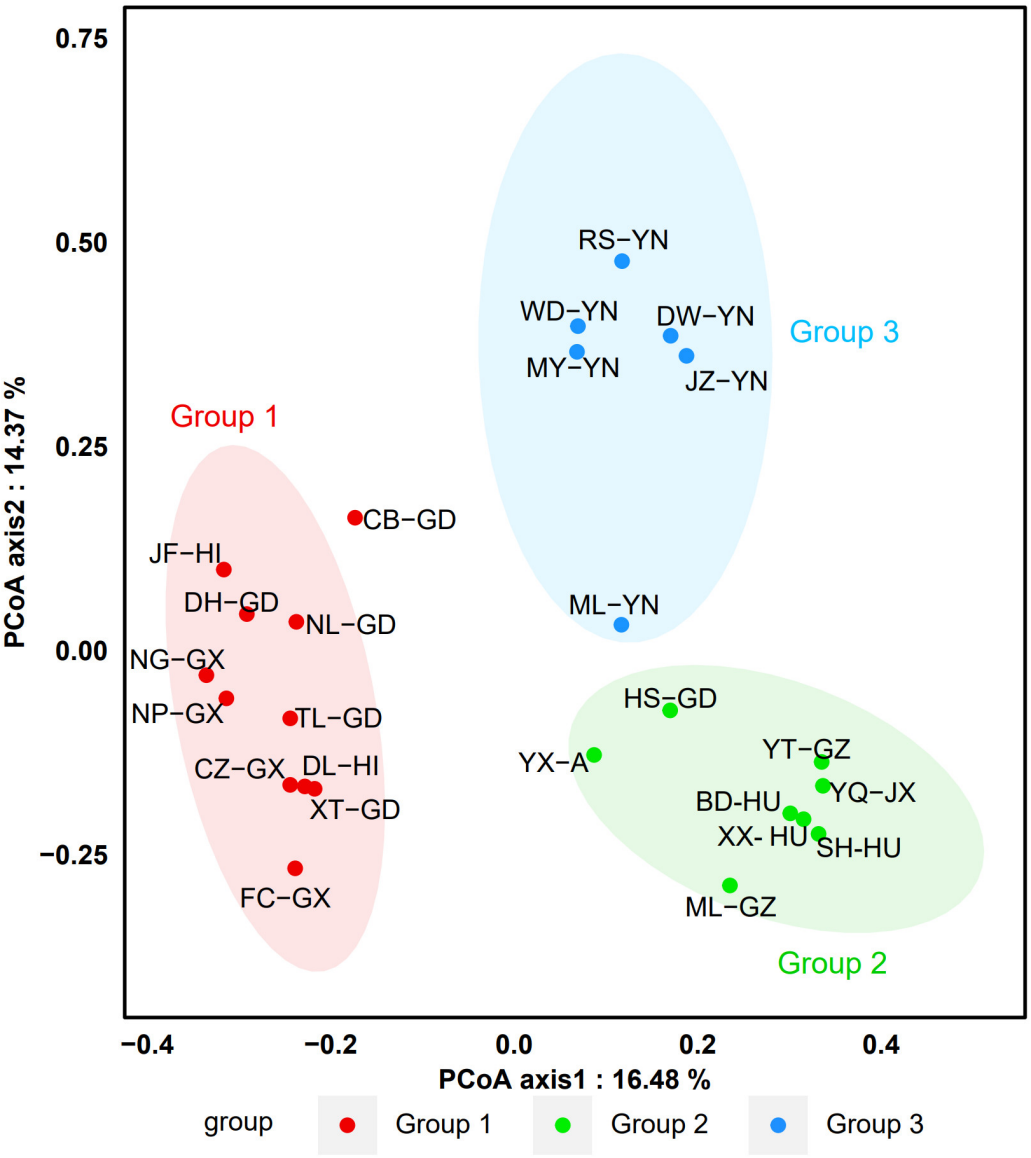
Principal coordinate analysis of 25 understory Passeriform communities in southern China. Points with the same colour represent the same group, Ellipses represent the 95% confidence interval around the group centroid.

The 25 communities were divided into three groups based on the clustering results of taxonomic diversity analysis. Group 1 included 11 sites in three southern China provinces (Guangdong, Guangxi, and Hainan). Group 2 included eight sites from five provinces in southeastern China (Jiangxi, Hunan, Guizhou, Anhui, and Guangdong). Group 3 included six sites from a single province in western China (Yunnan; Figure 3). Further, the PCoA results showed good agreement with the clustering results (Figure 4).

The PERMANOVA results revealed significant differences in taxonomic and functional β‐diversity between groups (Table 2). Interestingly, the sites in the same group colocalised in the same subregion (Figures 1 and 4). These results were also validated in terms of avian functional diversity. The taxonomic and functional β‐diversity showed geographical distribution patterns consistent with China's zoogeographical regions. Group 1 sites were mainly located in VIIA and VIIC. Group 2 sites were primarily in VIA and VIB, except for site HS‐GD, which was in an artificial forest. All sites in group 3 were located in VIIB (Figures 1 and 4).

**TABLE 2:** Permutational multivariate analysis of variance between three geographic groups based on taxonomic and functional β‐diversity.

Diversity	Group comparison	*R* ^2^	*p*
Taxonomic β‐diversity	PERMANOVA	.28	**<.001**
	Group 1 vs Group 2	.23	**.003**
	Group 1 vs Group 3	.23	**.003**
	Group 2 vs Group 3	.22	**.003**
Functional β‐diversity	PERMANOVA	.32	**<.001**
	Group 1 vs Group 2	.23	**.027**
	Group 1 vs Group 3	.25	**.006**
	Group 2 vs Group 3	.28	**.045**

*Note*: Bolded *p*‐values indicate a significant difference.

**FIGURE 5:**
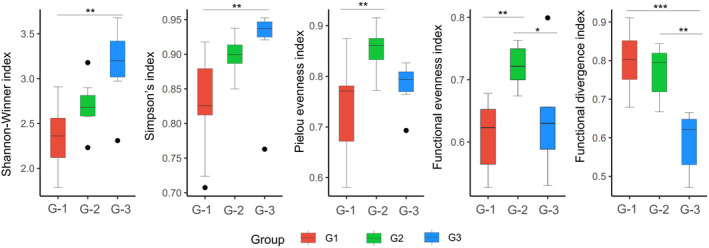
Comparison of diversity index between different groups. Letter code: G‐1 (Group 1), G‐2 (Group 2), G‐3 (Group 3). Asterisks indicate statistically significant differences between the two groups: “*” 0.05, “**” 0.01 and “***” 0.001.

**FIGURE 6:**
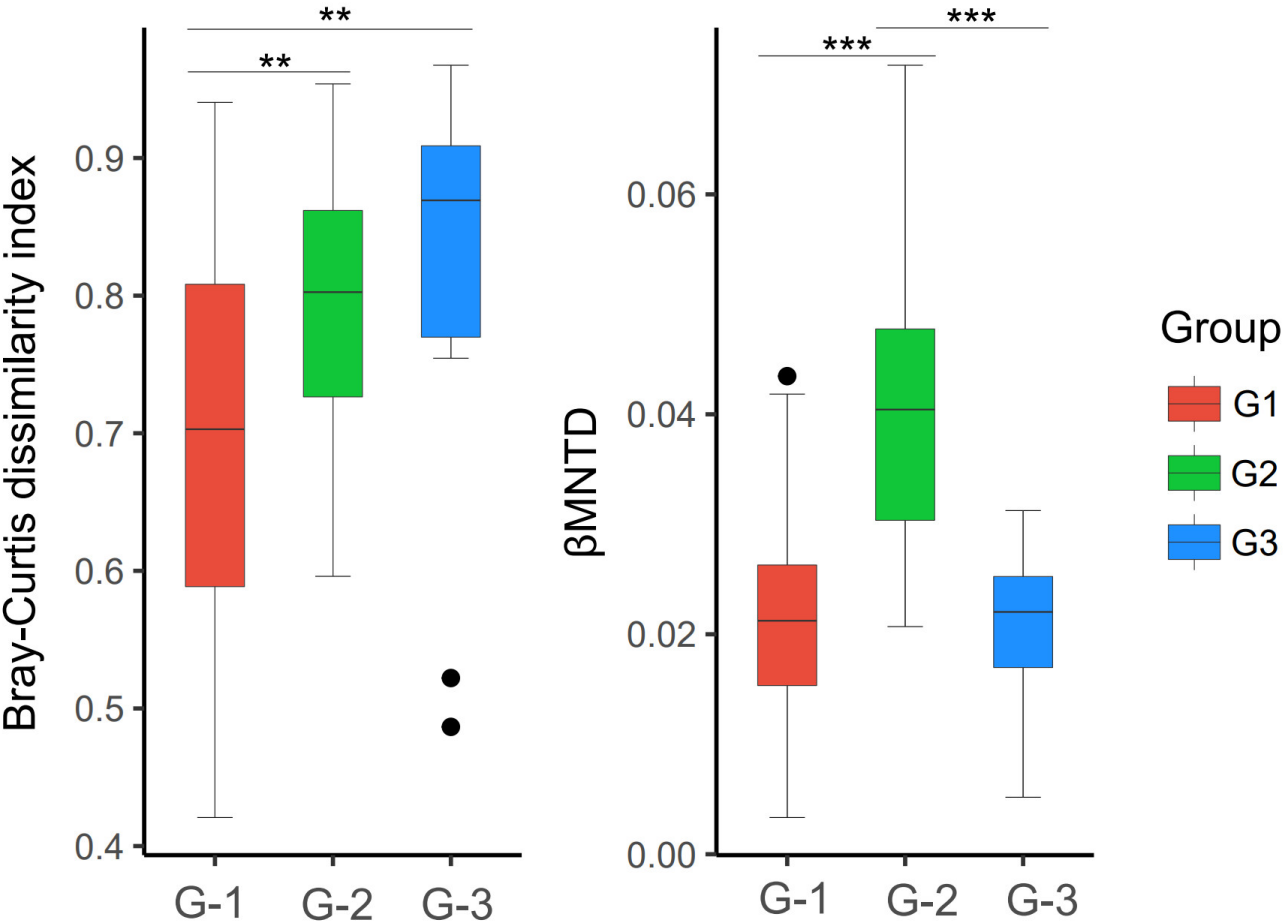
Comparison of taxonomic β‐diversity and functional β‐diversity between different groups. Letter code: G‐1 (Group 1), G‐2 (Group 2), G‐3 (Group 3). Asterisks indicate statistically significant differences between two groups: “**” 0.01 and “***” 0.001.

Group 3 in southwestern China exhibited the highest degree of diversity, in contrast to group 1 in southern China, which showed the lowest diversity. Based on the Shannon–Winner and Simpson indices, highly significant differences were noted between the two groups. However, the Pielou evenness index was the highest in group 2 and the lowest in group 1, with a highly significant difference between these groups. Additionally, group 2 showed the highest functional evenness compared to groups 1 and 3. Functional divergence was significantly higher in groups 1 and 2 than in group 3 (Figure 5). Groups 3 and 1 had the highest and lowest dissimilarity index, respectively, with a highly significant difference between the groups. Group 2 had the highest MNTD, with highly significant differences between groups 2 and 1, as well as between groups 2 and 3 (Figure 6).

### 3.3 Correlation between community and environmental factors

**FIGURE 7:**
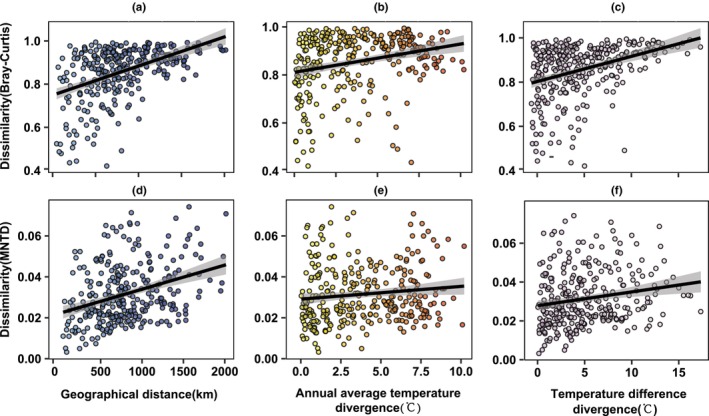
Relationships between understory Passeriformes bird taxonomic β‐diversity (a–c) and functional β‐diversity (d–f) and relevant factors (Geographical distance, annual mean temperature, and annual temperature range). Taxonomic β‐diversity and functional β‐diversity are quantified using the Bray–Curtis dissimilarity index and β mean nearest taxon distance, respectively. The grey band represents a ±95% confidence interval. We only present the relevant factors with significant values (*p* < .05).

We found significant correlations between both taxonomic and functional β‐diversity and geographical distance, AMT, and ATR (Figure 7 and Table 3). Nonetheless, no significant difference was observed between AMP or APR and taxonomic or functional β‐diversity (Table 3). Based on the results of an MRM analysis, geographical distance was identified as the most critical factor among the three significant correlation factors compared to AMT and ATR, which were Euclidean distance, both in terms of taxonomic and functional β‐diversity (please refer to Table S2).

**TABLE 3:** Mantel test between β‐diversity (taxonomic and functional) and correlation factors.

β‐Diversity	Correlation factors	*R*	*p*
Taxonomy	Geographical distance	0.44	**<.001**
Taxonomy	Annual mean temperature	0.25	**<.001**
Taxonomy	Annual Temperature range	0.37	**<.001**
Taxonomy	Annual mean precipitation	0.03	.626
Taxonomy	Annual Precipitation range	0.13	.079
Functional	Geographical distance	0.33	**<.001**
Functional	Annual mean temperature	0.15	**.020**
Functional	Annual Temperature range	0.21	**.026**
Functional	Annual mean precipitation	0.09	.234
Functional	Annual Precipitation range	0.11	.139

*Note*: Bolded *p*‐values are statistically significant.

## 4 DISCUSSION

Our study of understory birds was conducted in China and very few works were studied in the past. The study focused on the composition characteristics of understory bird communities and found what factors impact them. Our findings will provide robust support for the conservation of understory bird communities at the regional scale.

### 4.1 Characteristic of understory passeriformes bird community in southern China

We discovered that the understory bird community in southern China is predominantly comprised of Alcippeidae. The Huet's Fulvetta (*Alcippe hueti*) was particularly abundant, accounting for 37.9% of the total captures in primary tropical mountain rainforests and 29.5% in secondary tropical mountain rainforests in Hainan (Zou & Chen, 2004). This suggests that the Alcippeidae, Timaliidae, Leiothrichidae, and Pycnonotidae originated in mainland Asia (Cibois, 2003; Moyle et al., 2012). However, Xishuangbanna (Yunnan) exhibited a higher species richness and lower abundance, with the Sliver‐breasted Broadbill being the most frequently observed species, accounting for 12.7% of the species captured (Huang & Zou, 2010). This discrepancy may reflect the characteristics of avian community compositions in the two zoological regions, as their biological habitat is the primary determinant of the richness and abundance of understory bird communities (Young et al., 1998). In the Neotropics, suboscine songbirds of the Antbird (Thamnophilidae), Ovenbird (Furnariidae), Antthrush (Formicariidae), and Tyrant Flycatcher (Tyrannidae) families dominate understory insectivores (Powell, Cordeiro, & Stratford, 2015; Powell, Wolfe, et al., 2015). Common understory birds in northeast Tanzania include Broadbills, Greenbuls, Flycatchers, and Sunbirds (Mkongewa et al., 2013). In the eastern Himalayas, the six most prevalent species were Yellow‐throated Fulvetta (*Schoeniparus cinereus*), Rufous‐capped Babbler (*Cyanoderma ruficeps*), Rufouswinged Fulvetta (*Schoeniparus castaneceps*), Golden‐breasted Fulvetta (*Lioparus chrysotis*), Snowy‐browed Flycatcher (*Ficedula hyperythra*) and Golden Babbler (*Cyanoderma chrysaeum*) (Srinivasan et al., 2015).

The dominant understory bird species differed significantly between HS‐GD (artificial forest) and DH‐GD (natural forest). The former consisted primarily of the Great Tit (*Parus major*), Chinese Bulbul (*Pycnonotus sinensis*), and Red‐flanked Robin (*Tarsiger cyanurus*), while the latter mainly comprised Alcippeidae or Timaliidae. Migrants were frequently observed at the HS‐GD site, accounting for 42.9% of the total species and 33.6% of the total captures (Zou & Yang, 2005). These traits contrasted significantly with those of other understory bird communities in natural forests, where numerous species belonged to the Alcippeidae family. One possible explanation for the increased migration rate at the HS‐GD site could be the relatively young age of the forests, which are only 20 years old (Zou et al., 2014). Unfortunately, our study only included one artificial forest site; thus, we were unable to draw a robust conclusion on whether this phenomenon is common between artificial and natural forests in southern China. Similar observations have been reported for latitudinal migrants, which are more prevalent in younger habitats than in older forests (Petit et al., 1995; Robinson & Terborgh, 1997). Natural forests play a pivotal role in protecting endemic species.

### 4.2 The relationship between diversity patterns and zoogeographical zones

Our study, which focused on the taxonomic and functional β‐diversity of understory birds in southern China, revealed that the distribution patterns observed align with zoogeographical regionalisation (Zhang, 1999). The understory bird communities at the 25 sites were grouped into six sub‐regions within the Oriental Realm, implying that the distance between the sampling sites was a crucial factor in biogeographical regionalisation in our study. This phenomenon may be attributed to the limited dispersal ability of understory birds, highlighting a significant relationship between dissimilarity and geographic distance (Blake & Loiselle, 2009). The process of biogeographical regionalisation offers a spatial framework that aids in understanding historical events, ecological progress, and the establishment of conservation priorities (de Klerk et al., 2002). Consequently, our findings may indicate that species distribution is jointly determined by the evolutionary history and environmental factors (Wang et al., 2012). Kreft and Jetz (2010) developed a research framework to demarcate biogeographical regions based on the distributions of 256 species. Remarkably, our study is the first of its kind to examine the avian biogeographical regions specifically for understory birds. Our findings support the hypothesis that understory communities exhibit variation within and between regions, underscoring the need to consider the spatial scale of communities and develop comprehensive conservation plans aimed at preserving the full diversity of understory bird communities.

Overall, our findings indicate that temperature, including AMT and ATR, significantly influenced the distribution of understory birds. Conversely, precipitation, including AMP and APR, did not yield a significant impact. In addition to temperature, numerous other factors, such as species colonisation, competition, species physiology, and behavioural adaptations, play key roles in shaping species distribution and biogeographical regionalisation (Xie et al., 2004). He et al. (2017) examined the distribution patterns of amphibians, reptiles, birds, and non‐bat mammals in China and identified that AMP, temperature seasonality, and mean elevation were the most influential factors. Similarly, Ding et al. (2006) demonstrated a strong positive correlation between primary productivity and bird species richness. Multiple environmental variables seemingly contribute to determining understory bird communities.

## 5 CONCLUSION

Our results showed that the understory bird communities in the 25 sites located in southern China could be categorised into six sub‐regions within the Oriental Realm. The distribution patterns of β‐diversity, as determined by taxonomic and functional β‐diversity analyses, were consistent with zoogeographical regionalisation. The geographic distance, ATR, and AMT were the most significant factors associated with taxonomic and functional β‐diversity patterns. Consequently, we strongly advocate for the implementation of comprehensive conservation strategies that encompass the entire range of understory bird communities, taking into account both the spatial scale and the establishment of reserve systems.

## AUTHOR CONTRIBUTIONS

**Fangyuan Liu:** Formal analysis (equal); investigation (equal); software (equal); validation (equal); visualization (equal); writing – original draft (equal). **Xiaoping Yu:** Supervision (equal); writing – review and editing (equal). **Xianli Che:** Data curation (equal); methodology (equal). **Qiang Zhang:** Investigation (equal); supervision (equal); writing – review and editing (equal). **Alexandra Ashley Grossi:** Writing – review and editing (equal). **Min Zhang:** Investigation (equal); writing – review and editing (equal). **Zhengzhen Wang:** Data curation (equal); investigation (equal); writing – review and editing (equal). **Fasheng Zou:** Funding acquisition (equal); project administration (equal); supervision (equal); writing – review and editing (equal).

## CONFLICT OF INTEREST STATEMENT

The authors declare no conflict of interest.

## Supporting information


Tables S1–S2.

## Data Availability

The raw data that support the findings of this study is openly available at the Dryad Digital Repository (https://datadryad.org/stash/share/B4Pdc0VS5BrMMpWCE39L2QgSDId1q7w4BhwScUFXiHg).
