# Supplementary material for: Patterns and processes underlying understory songbird communities in southern China
Source: Ecol Evol. 2024 Jun 6;14(6):e11446. doi: 10.1002/ece3.11446 (PMC11154815; doi:10.1002/ece3.11446)
Supplement: Supplementary file 1 — Tables S1–S2. [file ECE3-14-e11446-s001.docx]

**TABLE S1** Environmental variables .

| Site | AMP(mm) | APR(mm) | AMT(℃） | ATR（℃） |
| --- | --- | --- | --- | --- |
| FC-GX | 1867.741 | 4649.333 | 21.85648 | 14.81111 |
| CZ-GX | 1404.25 | 4331.75 | 22.40833 | 15.99167 |
| XT-GD | 1643.233 | 3863.833 | 21.65451 | 13.85 |
| TL-GD | 1408.338 | 3331.25 | 21.45875 | 14.5 |
| NL-GD | 1523.737 | 3479.485 | 15.53187 | 18.72727 |
| HS-GD | 1189.88 | 3545.5 | 22.84 | 15.35 |
| DH-GD | 1597.015 | 3692.683 | 22.56783 | 14.64 |
| MY-YN | 1231.333 | 3139 | 20.7625 | 10.25 |
| ML-YN | 1499.775 | 4478 | 15.98583 | 12.8 |
| JZ-YN | 628 | 1317.5 | 15.17917 | 13.05 |
| WD-YN | 1292.616 | 3675.5 | 20.92535 | 11.5 |
| RS-YN | 1449.772 | 4406.638 | 19.87026 | 11 |
| DW-YN | 1197.133 | 3036.433 | 15.27 | 13.56667 |
| CB-GD | 1421.052 | 3871.25 | 18.77083 | 17.5 |
| YQ-JX | 1051.25 | 2244.625 | 18.175 | 26.5 |
| XX-HU | 1352.083 | 2899.9 | 17.12667 | 21.58 |
| SH-HU | 1262.75 | 2616.618 | 13.99804 | 20.78529 |
| BD-HU | 1197.989 | 2497.133 | 14.55556 | 22.18 |
| JF-HI | 1399.725 | 4012.167 | 21.74028 | 9.166667 |
| DL-HI | 1301.417 | 3764.5 | 24.08667 | 10.5 |
| YT-GZ | 806.375 | 1966.438 | 14.8875 | 23.56875 |
| ML-GZ | 1033.583 | 3363 | 18.45833 | 18.1 |
| NG-GX | 981.9167 | 2571.333 | 22.3125 | 14 |
| NP-GX | 1155.333 | 3515.556 | 21.48981 | 13.81111 |
| YX-AH | 1555.25 | 3041 | 13.89167 | 19.3 |

**TABLE S2** Result of the multiple regression on dissimilarity matrices analysis (MRM) for taxonomic and functional β-diversity.

|  | Taxonomic β-diversity  **R^2^=0.286, P<0.001** | | | Functional β-diversity  **R^2^=0.124, P<0.001** | |
| --- | --- | --- | --- | --- | --- |
|  | R^2^ | P | R2 | | P |
| Geographical distance | **0.069** | **<0.001** | **0.005** | | **0.001** |
| Annual mean temperature | **0.024** | **0.017** | 0.001 | | 0.786 |
| Annual temperature range | 0.020 | 0.142 | 0.000 | | 0.526 |
